# Sleep duration trajectories and all-cause mortality among Chinese elderly: A community-based cohort study

**DOI:** 10.1186/s12889-023-15894-3

**Published:** 2023-06-06

**Authors:** Rongxiu Ding, Pan Ding, Liuhong Tian, Xiaodan Kuang, Li Huang, Hongying Shi

**Affiliations:** 1 School and Hospital of Stomatology, Wenzhou Medical University, 325000 Wenzhou, Zhejiang P.R. China; 2 Department of Preventive Medicine, School of Public Health and Management, Wenzhou Medical University, 325000 Wenzhou, China

**Keywords:** Sleep duration trajectory, All-cause mortality, CLHLS, Chinese elderly

## Abstract

**Background:**

China is among the largest and fastest aging countries. The elderly population is more vulnerable, with higher proportion of inappropriate sleep duration and risk of mortality, compared with young and middle-aged adults. Single-measured sleep duration has been associated with mortality, but the health effects of long-term sleep duration trajectories remain unknown. This study aimed to explore the prospective associations between sleep duration trajectories and all-cause mortality among Chinese elderly.

**Methods:**

Participants (*n* = 3,895; median age: 82 years; females: 53.3%) who reported sleep duration in all three surveys (2005, 2008, and 2011) from the community-based Chinese Longitudinal Healthy Longevity Survey (CLHLS) were followed up until 2019 (about 8 years). We identified sleep duration trajectories by latent class mixed model and explored their association with all-cause mortality using Cox hazard proportional regression and Laplace regression models. Further, stratified analysis by demographic characteristics and lifestyles and sensitivity analysis by lag effect, health-related factors, and inverse probability weighting were used to verify the robustness of the association. In addition, we explored the threshold effect of baseline sleep duration on the risk of all-cause mortality.

**Results:**

We documented 1,881 all-cause deaths during 16,689 person-years of follow-up. Five sleep duration trajectories were identified: moderately increased trajectory (28.1%), rapidly increased trajectory (7.2%), persistent sleep trajectory of 7 h (33.7%), moderately decreased trajectory (21.3%), and rapidly decreased trajectory (9.7%). Compared with the persistent sleep trajectory of 7 h, the multivariable-adjusted *HRs* (95%*CI*) for moderately increased trajectory, rapidly increased trajectory, moderately decreased trajectory, and rapidly decreased trajectory were 1.21 (1.08, 1.36), 1.21 (1.01, 1.44), 0.95 (0.82, 1.10), and 0.93 (0.78, 1.11), respectively; and the corresponding difference in median survival time (95%*CI*) were -0.53 (-1.01, -0.05), -0.43 (0.16, -1.02), 0.26 (-0.34, 0.86), and 0.25 (-0.51, 1.02), respectively. Stratified and sensitivity analyses showed consistent results. Threshold analysis indicated a sharply increased risk of mortality in participants whose sleep exceeds 9 h (*HR* = 1.20, 95%*CI*: 1.11, 1.30).

**Conclusion:**

Compared with the persistent sleep trajectory of 7 h, moderately and rapidly increased sleep duration trajectories were associated with higher subsequent mortality in Chinese elderly. Those who report sleep exceeding 9 h may be at high risk for all-cause mortality.

**Supplementary Information:**

The online version contains supplementary material available at 10.1186/s12889-023-15894-3.

## Introduction

Recently, the proportion of the elderly population has accelerated. According to the United Nations report, 761 million (9.3%) people worldwide are 65 years old and above [1]. In China, 190 million (13.5%) people are 65 years old and above, making it one of the largest and fastest aging countries [2]. Unlike young and middle-aged adults, elderly people are more physically and mentally vulnerable and at higher risk for disease (e.g., stroke, cardiovascular disease, cancer) and mortality [3, 4], and therefore require more attention. Good sleep is involved in the self-repair and metabolism of organisms to promote health; while poor sleep may increase the risk of adverse health outcomes by influencing factors such as biological rhythms, and physical and psychological changes [5]. The sleep duration of the young and middle-aged population may be quite different from that of the elderly population, and the American Academy of Sleep Medicine’s recommendations for sleep duration varies by age group: 7-9 h for adults, and 7-8 h for elderly adults [6]. Studies have shown that older adults had a higher proportion of inappropriate sleep duration (≤ 6 or ≥ 9 h; 40.0–56.9% vs. 32.8–37.8%) [7–9] and a higher prevalence of multimorbidity (34.9–62.8% vs. 13.6–32.5%) [8–12] compared with young and middle-aged people. Therefore, more research among Chinese elderly could help develop targeted interventions and public health strategies to improve their overall health and well-being.

Previous studies have shown a U-shaped association between sleep duration and all-cause mortality [13], i.e., both long and short sleep duration increase the risk of all-cause mortality. However, most current studies only measured sleep duration once and did not capture the changes in sleep duration trajectory, which may more comprehensively reflect its impact on circadian rhythm [14]. The few available studies on sleep trajectories suggested that those with greater sleep variability or extreme long or short sleep are detrimental to physical and mental health [15–17], but there was little evidence between the sleep duration trajectory and the risk of all-cause mortality.

Some studies on sleep duration and mortality only considered linear differences in sleep duration between the two waves of surveys and reported a higher risk of all-cause mortality in older individuals with significant changes in sleep duration [18–20]. This assumption of purely linear variation was extremely sensitive to measurement error and prone to misclassification, leading to a bias in the true association between fluctuations in sleep duration and adverse health outcomes [15]. We found only one study explored the relationship between sleep trajectories and mortality among middle-aged adults, and their study found a higher mortality risk for sleep trajectories of persistent less than 5 h and decreasing from 7 to 5 h among 4 different sleep trajectories [21]. It is well known that the elderly population may have a significantly different sleep duration trajectory than middle-aged adults because their sleep duration usually decreases naturally with age [6, 22]. Therefore, the evidence for long-term sleep duration trajectories and risk of all-cause mortality among elderly adults is insufficient and further research is urgently needed.

In this study, based on the Chinese Longitudinal Survey of Healthy Longevity (CLHLS) database, we used the latent class mixed model from a life-course perspective to identify trajectories for three repeated measurements of sleep duration (2005, 2008, and 2011 year). And the association with subsequent all-cause mortality risk (until the 2019 year) was assessed after fully controlling for confounding factors such as demographic characteristics, lifestyle, and health information. Since sleep reduction with aging was a normal physiological phenomenon in the elderly, we hypothesized that participants with long sleep duration or with increased sleep duration trajectory had an increased risk of all-cause mortality compared to participants with persistent healthy sleep duration. In addition, we evaluated the association of sleep duration measured at baseline (the wave of the 2011 year) with the risk of all-cause mortality, and used threshold effects analysis to identify the appropriate sleep duration for the Chinese elderly.

## Methods

### Study design and participants

This study used data from the Chinese Longitudinal Survey of Healthy Longevity (CLHLS), an ongoing, open, community-based cohort established in 1998 [23–26]. During 1998–2018, the CLHLS conducted eight follow-up surveys in 23 provinces in China using a targeted, disproportionate sampling method to select approximately half of the counties and cities to obtain a sufficient number of older adults. The CLHLS collected information on demographic characteristics, lifestyle, and health status with follow-up every 3–4 years to understand the health risk factors in people aged 65 and over, especially those aged 80 and over [23]. The study was conducted through in-home, face-to-face interviews with trained physicians from community hospitals and public health workers from the Centers for Disease Control and Prevention. A previous study has shown that the non-response rate of CLHLS was very low, the data attrition (average per wave was 4.85%) was similar to that of large Western cohorts, and the data quality was generally good [27].

Information on sleep duration in the CLHLS database was collected from 2005; therefore, participants surveyed in 2005, 2008, and 2011 were potentially eligible for this study, and the baseline for this analysis was 2011 (e.g., the date when completing the 2011 survey). Of the 4,191 participants, we excluded 56 participants with missing sleep duration information from surveys in 2005, 2008, and 2011 waves, 25 participants with the missing date of death, and 215 participants without any follow-up, resulting in 3,895 participants included in the study (**eFig. 1**). We compared baseline characteristics (demographic characteristics, lifestyle, and health information) between the included and excluded populations and the result showed that no statistical differences were found for most baseline characteristics such as age, sex, marital status, education, economic status, body type, sleep quality, diet score, current exercise, self-rated health, cognitive function, depressive status, chronic disease, and hypertension; only a few baseline characteristics were found to be statistically different (*P* < 0.05): compared to the included population, a lower proportion of illiterates (53.6% vs. 46.3%), current drinkers (18.9% vs. 12.1%) and current smokers (19.5% vs. 12.5%) in the excluded population (**eTable 1**).

### Assessment of sleep duration

In three waves of surveys from 2005 to 2011, sleep duration within 24 h was assessed by the question “How long do you sleep normally including napping recently?”. Sleep duration within 24 h without differentiating between nighttime sleep duration and daytime naps is also often used in epidemiological studies [28], and studies have shown that nighttime sleep duration and sleep duration within 24 h have similar effects on health outcomes (e.g., all-cause mortality, dementia) [29, 30]. Previous studies have found a moderate correlation (*r* = 0.45–0.57) between subjective reports of habitual sleep and objective measured sleep (e.g., sleep logs, wrist actigraphy) [31–33], and its validity has been validated [33]. Considering the proportion of participants with sleep duration less than 5 h or more than 11 h was relatively low: 253 (6.5%), 214 (5.5%), and 326 (8.4%) people slept for less than 5 h in the 2005, 2008, and 2011 wave, respectively; 174 (4.5%), 208 (5.3%), and 337 (8.6%) people slept for more than 11 h in the 2005, 2008, and 2011 wave, respectively (**eFig. 2**), as well as referring to the grouping of sleep duration trajectories in previous studies [15, 21, 34], we divided sleep duration into 7 groups (≤ 5, 6, 7, 8, 9, 10, and ≥ 11 h).

### Outcome

We used all-cause mortality as an outcome indicator. During two follow-up visits (2014, 2018–2019), participants’ survival status and date of death were obtained from death certificates or interviews with next of kin, local resident councils, or community physicians [23–25].

### Assessment of covariates

The covariates were chosen based on previous studies in this field [24, 25] and were collected in 2011. Demographic characteristics information included sex (male, female), age, residence (city, town, rural), education (illiteracy, educated), occupation (farmers, others), marital status (married, others), economic status (rich, general, poor); health information included body type (underweight, normal, overweight), self-rated health (good, fair, poor), chronic disease (yes, no), cognitive function (normal, impairment), activities of daily living (normal, disabled), depression (yes, no), and hypertension (no, yes); lifestyle information included sleep quality (good, fair, poor), smoking status (never, former, current), drinking status (never, former, current), current exercise (no, yes), and diet score.

The body mass index was calculated based on weight (kg) divided by the square of the height (m). According to the Chinese standard [35], body type was divided into three categories: underweight (< 18.5 kg/m^2^), normal (18.5–23.9 kg/m^2^), and overweight (≥ 24 kg/m^2^). Chronic diseases included diseases with high incidence among the elderly in China [36]. Participants who self-reported one or more of the following conditions were considered as having chronic diseases: diabetes, heart disease, bronchitis, emphysema, pneumonia, asthma, stroke, or cerebrovascular disease [37]. Cognitive function was assessed by the Mini-mental State Examination (MMSE). The MMSE scale had 24 items, including general ability, attention and numeracy, reaction, memory, language comprehension, and self-coordination, with a total of 30 points, and was divided into cognitively normal (≥ 24 points) and impaired (< 24 points) [38]. Depressive status was assessed by the Center for Epidemiologic Studies Short Depression Scale (CES-D), which consisted of 7 items (each with a score between 0 and 4). The score of depression was 0 to 28 points, and the higher the score, the more serious the depression. Those with scores higher than 10 points were considered in a depressive state [39]. The dietary diversity score was assessed by 10 items: fresh fruits, vegetables, meat, fish, and other aquatic products, eggs, soy products, pickled vegetables, sugar, tea, and garlic. Responses for each item were always (1 point) to rarely (3 points), whereas responses for sugar and pickled vegetables were reversely coded before summing. The total score of the dietary pattern was 30 points, and the lower the score, the healthier the diet [26]. When information on the 2011 covariates was missing (missing values for occupation, education, and marital status were 6, 9, and 13 cases, respectively; missing rate < 5%), we filled in the data using data from the 2005 or 2008 surveys.

### Statistical analyses

We used the latent class mixed model, a group-based trajectory modeling approach (R, lcmm package), to identify subgroups with similar sleep duration trajectories during the three consecutive surveys from 2005 to 2011. Briefly, we fitted models with different numbers and forms of potential trajectories (linear, and quadratic slope) by maximum likelihood. Bayesian Information Criterion (BIC) and Entropy were used to determine the optimal form and number of the trajectory. Based on a previous study [40], the proportion of participants in each trajectory should be at least 5% of the population. Finally, we also tested discriminative ability by the posterior probability of group membership.

The Chi-square test was used for comparing unordered categorical data and the Kruskal-Wallis test was used for ordinal data and nonnormal continuous data. We used Kaplan-Meier analysis to assess the survival trends in different trajectories of sleep duration. Person-years were calculated from the date of completion of the questionnaire in 2011 to the date of death, loss to follow-up, or the end of follow-up (July 31, 2019), whichever came first. We assessed the association between trajectories of sleep duration and all-cause mortality using Cox proportional hazards regression and Laplace regression models. Laplace regression complements Cox regression analysis, which estimates differences in time to experience death events. Results are expressed as hazard ratios (*HRs*) and 95% confidence intervals (*CIs*) and differences (95%*CI*) in median survival time. The MV1 model was adjusted for demographic information (sex, age, place of residence, education, occupation, economic status, and marital status). The MV2 model was further adjusted for health information (self-rated health, chronic disease, depression, cognitive function, activities of daily living, and hypertension). The MV3 model was further adjusted for lifestyle factors (body type, drinking status, smoking status, current exercise, sleep quality, and diet score). Schoenfeld’s residuals test was used to evaluate the proportional hazards (PH) assumption. Further, we performed the stratified analysis by age, sex, body type, smoking status, drinking status, current exercise, and sleep quality. And we also used likelihood ratio tests to assess statistical interactions between variables for comparing models with and without multiplicative interaction terms. For comparison with previous studies, we assessed the association of sleep duration at baseline (the wave of 2011) with the risk of all-cause mortality by restricted spline curve and threshold effect analysis.

Several sensitivity analyses were used to verify the robustness of the results. Firstly, we considered a two-year lag analysis to address potential reverse causation by excluding older adults who died within two years after baseline. Secondly, we restricted participants to good self-rated health, normal cognitive function, no depression, no chronic disease, and no hypertension, and reassessed the association of sleep duration trajectories with all-cause mortality. Thirdly, to further enhance the comparability between different trajectory groups, we also assessed the association between sleep duration trajectories and all-cause mortality by inverse probability weighting. Finally, based on previous studies [23, 41], we reassessed the association of sleep duration trajectories with all-cause mortality after excluding those participants who reported extreme sleep duration (< 3 or > 16 h; excluded if reported once in all three waves).

All statistical analyses were implemented using Empower (R) (www.empowerstats.com, X & Y solutions, inc. Boston MA) and R (http://www.R-project.org). A 2-sided *P* value < 0.05 was considered statistically significant.

## Results

The median (*IQR*) age of the 3,895 elderly was 82 (76, 91) years, and 53.3% of them were female. There was 1,881 death during the 16,689 person-years of the follow-up period (median follow-up was 3.5 years).

### Trajectories of sleep duration in the elderly from 2005 to 2011

**Fig. 1:**
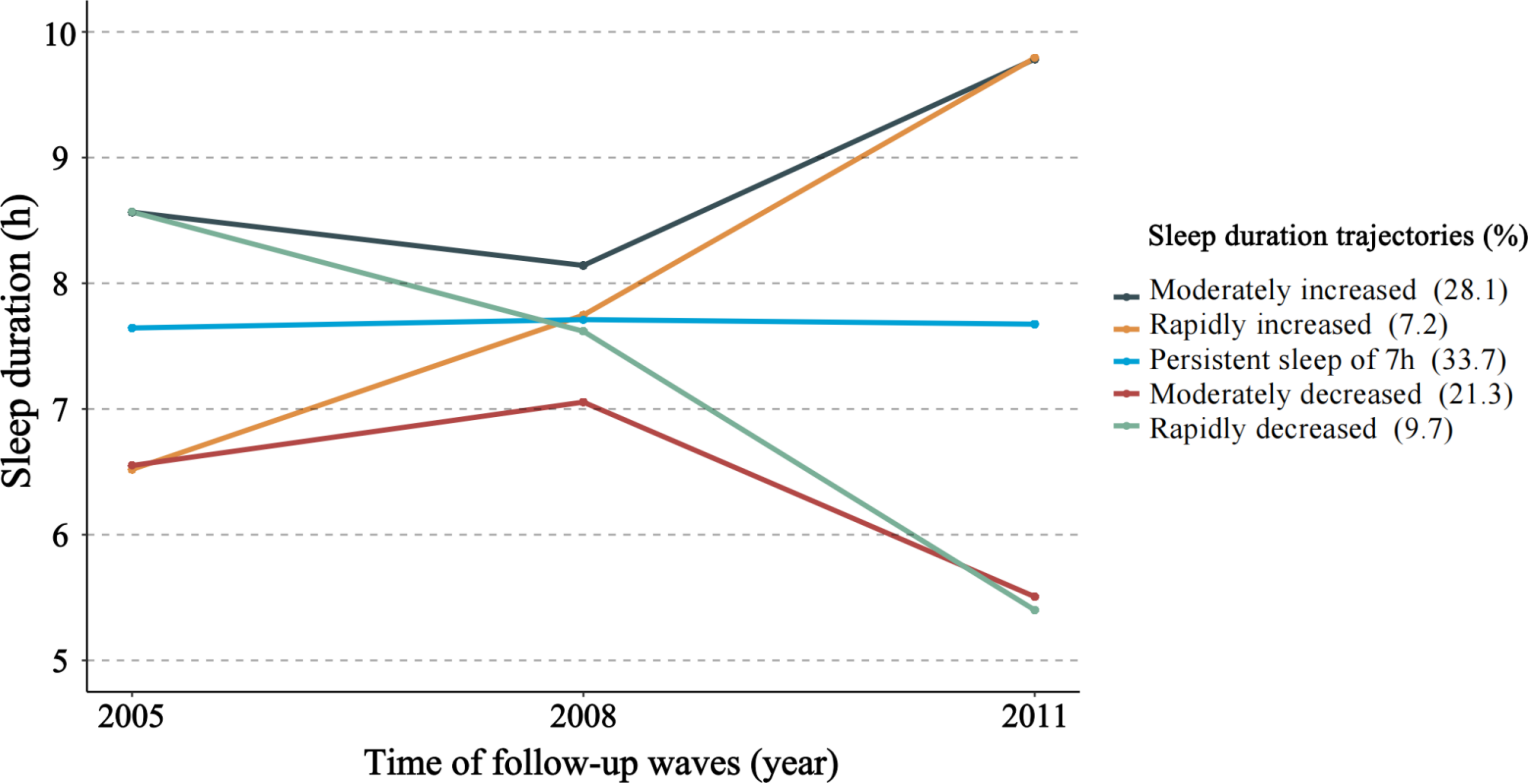
Sleep duration trajectories from 2005 to 2011 in the Chinese elderly

Using the latent class mixed model, we identified 5 different sleep trajectories in older adults from 2005 to 2011 (**eTable 2**): 1,095 (28.1%) people have increased sleep duration from 8 to 9 h (moderately increased trajectory), 279 (7.2%) people have increased sleep duration from 6 to 9 h (rapidly increased trajectory), 1,313 (33.7%) people have a persistent sleep duration of 7 h (persistent sleep trajectory of 7 h), 832 (21.3%) people have decreased sleep duration from 7 to 5 h (moderately decreased trajectory), 376 (9.7%) people have decreased sleep duration from 8 to 5 h (rapidly decreased trajectory) (Fig. 1). We calculated the posterior probability of each individual being a member of 5 trajectory groups and assigned them to the trajectory group with the largest posterior probability of membership. The posterior probabilities of the 5 trajectory groups were all greater than 0.70 (0.81, 0.76, 0.99, 0.83, and 0.71, respectively). This suggests that each trajectory we fitted has high internal reliability and sufficient discrimination among participants with different sleep duration trajectories.

### Baseline characteristics

Table 1 showed the baseline characteristics by sleep trajectory groups. Compared with those who had persistent sleep trajectory of 7 h, those with sleep duration change (those who had decreased sleep trajectory or those who had increased sleep trajectory) were older, had a higher proportion of females, farmers, illiterates, poor economic status, underweight, unhealthy diet, poor sleep quality, never smokers, current exercisers, and poor health status (self-rated poor health status, cognitive impairment, depression). In addition, the proportion of chronic diseases and hypertension was higher in the moderately decreased trajectory. All *P* < 0.05.

**Table 1:** Characteristics of participants according to sleep duration trajectories

Characteristics	Sleep duration trajectories					*P*-value
	Moderately increased (*n* = 1,095)	Rapidly increased (*n* = 279)	Persistent sleep of 7 h (*n* = 1,313)	Moderately decreased (n = 832)	Rapidly decreased (n = 376)	
**Female**, ***n*(%)**	573 (52.3)	154 (55.2)	638 (48.6)	496 (59.6)	216 (57.4)	
**Age, years, median (** ***IQR*** **)**	86.0 (78.0, 94.0)	85.0 (78.0, 93.0)	80.0 (75.0, 88.0)	80.0 (75.0, 86.0)	83.0 (76.0, 91.0)	**< 0.001**
**Place of residence**, ***n*(%)**						**< 0.001**
City	189 (17.3)	58 (20.8)	314 (23.9)	194 (23.3)	46 (12.2)	
Town	384 (35.1)	90 (32.3)	459 (35.0)	295 (35.5)	156 (41.5)	
Rural	522 (47.7)	131 (47.0)	540 (41.1)	343 (41.2)	174 (46.3)	
**Illiteracy**, ***n*(%)**	625 (57.1)	165 (59.1)	640 (48.7)	439 (52.8)	218 (58.0)	**< 0.001**
**Farmers**, ***n*(%)**	764 (69.8)	194 (69.5)	816 (62.1)	516 (62.0)	282 (75.0)	**< 0.001**
**Economic status**, ***n*(%)**						**< 0.001**
Rich	204 (18.6)	58 (20.8)	260 (19.8)	136 (16.3)	53 (14.1)	
General	707 (64.6)	175 (62.7)	857 (65.3)	528 (63.5)	230 (61.2)	
Poor	184 (16.8)	46 (16.5)	196 (14.9)	168 (20.2)	93 (24.7)	
**Married**, ***n*(%)**	411 (37.5)	91 (32.6)	621 (47.3)	358 (43.0)	135 (35.9)	**< 0.001**
**Sleep quality**, ***n*(%)**						**< 0.001**
Good	886 (80.9)	204 (73.1)	941 (71.7)	269 (32.3)	104 (27.7)	
Fair	152 (13.9)	49 (17.6)	288 (21.9)	277 (33.3)	131 (34.8)	
Poor	57 (5.2)	26 (9.3)	84 (6.4)	286 (34.4)	141 (37.5)	
**Drinking status**, ***n*(%)**						0.157
Never	692 (63.2)	181 (64.9)	804 (61.2)	555 (66.7)	249 (66.2)	
Former	183 (16.7)	46 (16.5)	241 (18.4)	147 (17.7)	61 (16.2)	
Current	220 (20.1)	52 (18.6)	268 (20.4)	130 (15.6)	66 (17.6)	
**Smoking status**, ***n*(%)**						**0.029**
Never	691 (63.1)	171 (61.3)	769 (58.6)	527 (63.3)	236 (62.8)	
Former	212 (19.4)	42 (15.1)	261 (19.9)	164 (19.7)	64 (17.0)	
Current	192 (17.5)	66 (23.7)	283 (21.6)	141 (16.9)	76 (20.2)	
**Current exercise**, ***n*(%)**	627 (47.8)	394 (36.0)	102 (36.6)	372 (44.7)	118 (31.4)	**< 0.001**
**Diet score, median (** ***IQR*** **)**	22.0 (20.0, 24.0)	23.0 (20.0, 25.0)	23.0 (20.0, 25.0)	23.0 (20.0, 25.0)	22.0 (19.0, 24.0)	**< 0.001**
**Self-rated health**, ***n*(%)**						**< 0.001**
Good	530 (48.4)	128 (45.9)	632 (48.1)	299 (35.9)	131 (34.8)	
Fair	377 (34.4)	88 (31.5)	477 (36.3)	311 (37.4)	133 (35.4)	
Poor	188 (17.2)	63 (22.6)	204 (15.5)	222 (26.7)	112 (29.8)	
**Body type**, ***n*(%)**						**< 0.001**
Underweight	319 (29.1)	96 (34.4)	308 (23.5)	206 (24.8)	124 (33.0)	
Normal	590 (53.9)	138 (49.5)	712 (54.2)	450 (54.1)	188 (50.0)	
Overweight	186 (17.0)	45 (16.1)	293 (22.3)	176 (21.2)	64 (17.0)	
**Cognitive impairment**, ***n*(%)**	497 (45.4)	126 (45.2)	381 (29.0)	251 (30.2)	159 (42.3)	**< 0.001**
**Depression**, ***n*(%)**	447 (40.8)	126 (45.2)	445 (33.9)	315 (37.9)	186 (49.5)	**< 0.001**
**Chronic disease**, ***n*(%)**	389 (35.5)	110 (39.4)	516 (39.3)	370 (44.5)	133 (35.4)	**0.001**
**Hypertension**, ***n*(%)**	404 (36.9)	100 (35.8)	528 (40.2)	365 (43.9)	142 (37.8)	**0.017**

Moderately increased trajectory (from 8 to 9 h), rapidly increased trajectory (from 6 to 9 h), moderately decreased trajectory (from 7 to 5 h), rapidly decreased trajectory (from 8 to 5 h)

The Chi-square test was used for comparing unordered categorical data and the Kruskal-Wallis test was used for ordinal data and nonnormal continuous data, and bold values indicated statistical significance *P* < 0.05

The missing values for occupation, education, and marital status were 6, 9, and 13, respectively

### Trajectories of sleep duration and risk of all-cause mortality

The survival curve for participants showed 5 sleep duration trajectories (moderately increased trajectory, rapidly increased trajectory, persistent sleep trajectory of 7 h, moderately decreased trajectory, and rapidly decreased trajectory; **eFig. 3**) with median survival times of 4.47, 4.71, 7.75, 7.29, and 6.14 years, respectively; log-rank *P* < 0.001.

In the age-adjusted model, compared with the persistent sleep trajectory of 7 h, the *HR* (95%*CI*) for all-cause mortality was 1.23 (1.09, 1.38) for the moderately increased trajectory, 1.28 (1.07, 1.52) for the rapidly increased trajectory, 1.01 (0.88, 1.15) for the moderately decreased trajectory, and 1.05 (0.89, 1.25) for the rapidly decreased trajectory. Compared with the persistent sleep trajectory of 7 h, the Laplace regression showed that the median survival time (95%*CI*) was -0.72 years (-1.20, -0.23) for the moderately increased trajectory, -0.99 years (-1.62, -0.36) for the rapidly increased trajectory, 0.02 years (-0.66, 0.71) for the moderately decreased trajectory, and -0.25 years (-1.06, 0.56) for the rapidly decreased trajectory. After adjusting for demographic characteristic confounders (MV1), the association remained unchanged: compared with the persistent sleep trajectory of 7 h, the *HRs* (95%*CI*) were 1.23 (1.09, 1.38), 1.28 (1.07, 1.53), 1.03 (0.90, 1.18), and 1.05 (0.88, 1.24) for moderately increased trajectory, rapidly increased trajectory, moderately decreased trajectory, and rapidly decreased trajectory, respectively; and the corresponding difference in median survival time (95%*CI*) was -0.78 years (-1.30, -0.26), -0.77 years (-1.36, -0.18), -0.01 years (-0.65, 0.62), and -0.05 years (-0.88, 0.78), respectively. The association between increased sleep trajectory and all-cause mortality remained statistically significant after additional adjustment for health-related factors (MV2): the *HRs* (95%*CI*) were 1.22 (1.09, 1.37), 1.23 (1.03, 1.46), 0.96 (0.84, 1.10), and 0.96 (0.81, 1.14) for moderately increased trajectory, rapidly increased trajectory, moderately decreased trajectory, and rapidly decreased trajectory, respectively; and the corresponding difference in median survival time (95%*CI*) was -0.61 years (-1.08, -0.14), -0.74 years (-1.32, -0.17), 0.04 years (-0.53, 0.60), and -0.02 years (-0.81, 0.76), respectively. After further controlling for confounding by lifestyle confounders (MV3), we found that the association was weaker: compared with the persistent sleep trajectory of 7 h, the multivariable-adjusted *HRs* (95%*CI*) were 1.21 (1.08, 1.36), 1.21 (1.01, 1.44), 0.95 (0.82, 1.10), and 0.93 (0.78, 1.11) for moderately increased trajectory, rapidly increased trajectory, moderately decreased trajectory, and rapidly decreased trajectory, respectively; and the corresponding difference in median survival time (95%*CI*) was -0.53 years (-1.01, -0.05), -0.43 years (0.16, -1.02), 0.26 years (-0.34, 0.86), and 0.25 years (-0.51, 1.02), respectively. (Table 2).

**Table 2:** *HRs* (95%*CI*) of all-cause mortality and median survival time according to sleeping duration trajectories

Sleep duration trajectories		Age-adjusted	MV1	MV2	MV3
	Cases/person-years	Adjusted *HRs* (95%*CI*) *P*-value			
**Moderately increased**	648/4,265	**1.23 (1.09, 1.38) 0.001**	**1.23 (1.09, 1.38) 0.001**	**1.22 (1.09, 1.37) 0.001**	**1.21 (1.08, 1.36) 0.001**
**Rapidly increased**	164/1,081	**1.28 (1.07, 1.52) 0.006**	**1.28 (1.07, 1.53) 0.006**	**1.23 (1.03, 1.46) 0.023**	**1.21 (1.01, 1.44) 0.033**
**Persistent sleep of 7 h**	542/5,910	1.00	1.00	1.00	1.00
**Moderately decreased**	346/3,789	1.01 (0.88, 1.15) 0.917	1.03 (0.90, 1.18) 0.714	0.96 (0.84, 1.10) 0.595	0.95 (0.82, 1.10) 0.500
**Rapidly decreased**	181/1,644	1.05 (0.89, 1.25) 0.522	1.05 (0.88, 1.24) 0.589	0.96 (0.81, 1.14) 0.629	0.93 (0.78, 1.11) 0.448
**median survival time (years)**		**Differences (95%** ***CI*** **) in median survival time (years)** ***P*** **-value**			
**Moderately increased**	4.47	**-0.72 (-1.20, -0.23) 0.004**	**-0.78 (-1.30, -0.26) 0.003**	**-0.61 (-1.08, -0.14) 0.011**	**-0.53 (-1.01, -0.05) 0.030**
**Rapidly increased**	4.71	**-0.99 (-1.62, -0.36) 0.002**	**-0.77 (-1.36, -0.18) 0.010**	**-0.74 (-1.32, -0.17) 0.011**	-0.43 (0.16, -1.02) 0.150
**Persistent sleep of 7 h**	7.75	0.00	0.00	0.00	0.00
**Moderately decreased**	7.29	0.02 (-0.66, 0.71) 0.950	-0.01 (-0.65, 0.62) 0.967	0.04 (-0.53, 0.60) 0.899	0.26 (-0.34, 0.86) 0.393
**Rapidly decreased**	6.14	-0.25 (-1.06, 0.56) 0.550	-0.05 (-0.88, 0.78) 0.912	-0.02 (-0.81, 0.76) 0.959	0.25 (-0.51, 1.02) 0.518

Abbreviation: HR, hazard ratio; CI, confidence interval

Moderately increased trajectory (from 8 to 9 h), rapidly increased trajectory (from 6 to 9 h), moderately decreased trajectory (from 7 to 5 h), rapidly decreased trajectory (from 8 to 5 h)

MV1 model was adjusted for sex (male, female), age (continuous), place of residence (city, town, rural), education (illiteracy, educated), occupation (farmers, others), economic status (rich, general, poor), marital status (married, others). MV2 model was further adjusted for self-rated health (good, fair, poor), chronic disease (no, yes), depression (no, yes), cognitive function (normal, impairment), hypertension (no, yes). MV3 model was further adjusted for drinking status (never, former, current), smoking status (never, former, current), current exercise (no, yes), sleep quality (good, fair, poor), diet score (continuous), body type (underweight, normal, overweight)

The bold values indicated statistical significance *P* < 0.05

### Stratified and sensitivity analyses

In stratified analyses, the association of sleep duration trajectories and risk of all-cause mortality were consistent in subgroups with different ages, sex, body type, smoking status, drinking status, current exercise, and sleep quality, all *P* for interaction > 0.05 (Table 3).

**Table 3:** Association between sleep duration trajectories and the risk of all-cause mortality, stratification analyses

Subgroups	*n*	Cases/person-years	Sleep duration trajectories, *HRs (95%*CI*) *P*-value					*P* for interaction
			Moderately increased	Rapidly increased	Persistent sleep of 7 h	Moderately decreased	Rapidly decreased	
**Age, years**								0.564
≤ 80	1,735	455/9,136	**1.40 (1.10, 1.77) 0.006**	1.17 (0.79, 1.74) 0.426	1.00	0.83 (0.63, 1.09) 0.183	0.82 (0.57, 1.17) 0.278	
> 80	2,160	1426/7,553	**1.29 (1.13, 1.48) < 0.001**	**1.30 (1.07, 1.59) 0.009**	1.00	0.89 (0.75, 1.06) 0.203	0.89 (0.73, 1.09) 0.265	
**Sex**								0.184
Male	1,818	903/7,746	**1.28 (1.08, 1.50) 0.004**	**1.31 (1.01, 1.70) 0.043**	1.00	0.96 (0.77, 1.18) 0.675	1.05 (0.80, 1.37) 0.728	
Female	2,077	978/8,943	1.13 (0.95, 1.34) 0.154	1.13 (0.88, 1.44) 0.332	1.00	0.94 (0.77, 1.15) 0.522	0.85 (0.67, 1.09) 0.203	
**Body Type**								0.122
Underweight	1,053	664/3,865	**1.36 (1.11, 1.66)** **0.003**	1.30 (0.98, 1.72) 0.070	1.00	0.78 (0.60, 1.00) 0.052	0.94 (0.70, 1.25) 0.654	
Normal	2,078	956/9,119	**1.31 (1.11, 1.53) 0.001**	1.16 (0.89, 1.50) 0.279	1.00	0.93 (0.76, 1.14) 0.485	0.78 (0.60, 1.01) 0.062	
Overweight	764	261/3,705	1.32 (0.94, 1.84) 0.104	**2.17 (1.33, 3.55) 0.002**	1.00	1.11 (0.76, 1.63) 0.587	1.33 (0.82, 2.15) 0.246	
**Smoking Status**								0.093
Never	2,394	1,123/10,444	**1.28 (1.10, 1.48) 0.002**	1.15 (0.92, 1.45) 0.226	1.00	0.85 (0.70, 1.03) 0.092	**0.77 (0.61, 0.97) 0.025**	
Former	743	403/2,938	**1.43 (1.12, 1.84) 0.006**	**2.04 (1.32, 3.15) 0.001**	1.00	0.90 (0.66, 1.23) 0.504	1.40 (0.94, 2.08) 0.097	
Current	758	355/3,307	**1.27 (1.00, 1.67)** **0.049**	1.24 (0.84, 1.82) 0.273	1.00	0.92 (0.65, 1.29) 0.623	0.81 (0.54, 1.21) 0.299	
**Drinking Status**								0.151
Never	2,481	1,196/10,624	**1.29 (1.11, 1.49) 0.001**	1.18 (0.95, 1.48) 0.143	1.00	0.89 (0.74, 1.07) 0.208	0.81 (0.65, 1.01) 0.062	
Former	678	363/2,718	1.26 (0.96, 1.65) 0.101	**1.54 (1.01, 2.36) 0.045**	1.00	0.82 (0.58, 1.14) 0.231	1.09 (0.71, 1.66) 0.698	
Current	736	322/3,348	1.17 (0.88, 1.55) 0.286	1.20 (0.78, 1.84) 0.400	1.00	0.92 (0.63, 1.34) 0.659	1.02 (0.66, 1.56) 0.941	
**Current Exercise**								0.076
No	2,282	1,253/9,134	**1.24 (1.07, 1.44) 0.004**	1.19 (0.96, 1.48) 0.114	1.00	0.86 (0.71, 1.03) 0.098	**0.77 (0.62, 0.96) 0.018**	
Yes	1,613	628/7,555	**1.22 (1.00, 1.49) 0.050**	1.29 (0.94, 1.76) 0.115	1.00	0.98 (0.77, 1.25) 0.893	1.22 (0.88, 1.68) 0.241	
**Sleep Quality**								0.325
Good	2,404	1,146/10,365	**1.24 (1.08, 1.42) 0.002**	1.15 (0.93, 1.42) 0.184	1.00	0.84 (0.67, 1.06) 0.145	0.85 (0.62, 1.15) 0.298	
Fair	897	437/3,754	**1.31 (1.00, 1.73) 0.049**	**1.52 (1.01, 2.28) 0.046**	1.00	0.97 (0.75, 1.26) 0.818	0.96 (0.70, 1.30) 0.773	
Poor	594	298/2,570	1.19 (0.74, 1.93) 0.467	1.05 (0.56, 1.95) 0.885	1.00	0.85 (0.60, 1.22) 0.388	0.85 (0.57, 1.27) 0.425	

Abbreviation: HR, hazard ratio; CI, confidence interval

Moderately increased trajectory (from 8 to 9 h), rapidly increased trajectory (from 6 to 9 h), moderately decreased trajectory (from 7 to 5 h), rapidly decreased trajectory (from 8 to 5 h)

*Adjusted for sex (male, female), age (≤ 80, > 80 years), place of residence (city, town, rural), education (illiteracy, educated), occupation (farmers, others), economic status (rich, general, poor), marital status (married, others), self-rated health (good, fair, poor), chronic disease (no, yes), depression (no, yes), cognitive function (normal, impairment), activities of daily living (normal, disabled), hypertension (no, yes), body type (underweight, normal, overweight), drinking status (never, former, current), smoking status (never, former, current), current exercise (no, yes), sleep quality (good, fair, poor), diet score (continuous), except for the stratification factor itself

The bold values indicated statistical significance *P* < 0.05

We performed several sensitivity analyses. Firstly, when we excluded older adults who died within two years of follow-up, we found a similar association of sleep duration trajectories with all-cause mortality risk. Secondly, to further control the confounding of healthy factors, we restricted participants to good self-rated health, normal cognitive function, no depression, no chronic disease, and no hypertension, the association between sleep duration trajectories and the risk of all-cause mortality was consistent. Thirdly, We also assessed the association of sleep trajectories with the risk of all-cause mortality using inverse probability weighting and found consistent results. Finally, the association of sleep duration trajectories with all-cause mortality remained robust after those participants who reported extreme sleep duration were excluded (Table 4).

**Table 4:** Sensitivity analyses of the association between trajectories of sleep duration and the risk of all-cause mortality

Outcomes	*n*	Cases/person-years	Sleep duration trajectories, *HRs* (95%*CI*) *P*-value				
			Moderately increased	Rapidly increased	Persistent sleep of 7 h	Moderately decreased	Rapidly decreased
1. ^a^ Excluded of participants who died within two-years of follow-up							
	3,091	1,078/15,860	**1.31 (1.13, 1.53) 0.001**	1.13 (0.89, 1.46) 0.303	1.00	0.96 (0.80, 1.15) 0.655	0.84 (0.66, 1.07) 0.154
**2.**^**a**^**Limiting population to**:							
Good self-rated health	1,720	757/7,670	**1.39 (1.16, 1.66) < 0.001**	1.19 (0.90, 1.59) 0.208	1.00	0.82 (0.64, 1.05) 0.109	0.93 (0.69, 1.25) 0.630
Normal cognitive function	2,481	907/11,997	**1.27 (1.07, 1.50) 0.006**	1.12 (0.86, 1.47) 0.396	1.00	0.93 (0.76, 1.13) 0.453	1.00 (0.77, 1.28) 0.977
No depression	2,376	951/11,091	**1.25 (1.06, 1.48) 0.007**	1.14 (0.88, 1.48) 0.308	1.00	0.92 (0.75, 1.12) 0.417	0.98 (0.74, 1.28) 0.861
No chronic disease	2,377	1,121/10,546	**1.21 (1.04, 1.41) 0.014**	**1.32 (1.05, 1.67) 0.018**	1.00	0.94 (0.78, 1.14) 0.559	0.90 (0.72, 1.13) 0.362
No hypertension	2,356	1,176/9,997	**1.34 (1.15, 1.55) < 0.001**	**1.39 (1.11, 1.73) 0.003**	1.00	0.86 (0.71, 1.04) 0.124	0.90 (0.73, 1.12) 0.364
**3.** ^**b**^ **Inverse probability weighting**							
	3,895	1,881/16,689	**1.21 (1.06, 1.38) 0.006**	1.13 (0.91, 1.40) 0.261	1.00	0.86 (0.74, 1.01) 0.066	0.91 (0.73, 1.13) 0.379
**4.** ^**a**^ **Excluded those participants who reported extreme sleep duration (< 3 or > 16 h)**							
	3,591	1,727/15,407	**1.22 (1.09, 1.38**) **0.001**	**1.23 (1.02, 1.48) 0.032**	1.00	0.96 (0.82, 1.12) 0.579	0.98 (0.81, 1.18) 0.824

Abbreviation: HR, hazard ratio; CI, confidence interval

Moderately increased trajectory (from 8 to 9 h), rapidly increased trajectory (from 6 to 9 h), moderately decreased trajectory (from 7 to 5 h), rapidly decreased trajectory (from 8 to 5 h)

^a^ Adjusted for sex (male, female), age (continuous), place of residence (city, town, rural), education (illiteracy, educated), occupation (farmers, others), economic status (rich, general, poor), marital status (married, others), self-rated health (good, fair, poor), chronic disease (no, yes), depression (no, yes), cognitive function (normal, impairment), activities of daily living (normal, disabled), hypertension (no, yes), body type (underweight, normal, overweight), drinking status (never, former, current), smoking status (never, former, current), current exercise (no, yes), sleep quality (good, fair, poor), diet score (continuous)

^b^ Propensity weight was based on: sex (male, female), age (continuous), place of residence (city, town, rural), education (illiteracy, educated), occupation (farmers, others), economic status (rich, general, poor), marital status (married, others), self-rated health (good, fair, poor), chronic disease (no, yes), depression (no, yes), cognitive function (normal, impairment), activities of daily living (normal, disabled), hypertension (no, yes), body type (underweight, normal, overweight), drinking status (never, former, current), smoking status (never, former, current), current exercise (no, yes), sleep quality (good, fair, poor), diet score (continuous)

The bold values indicated statistical significance *P* < 0.05

### Sleep duration at baseline and all-cause mortality

**Fig. 2:**
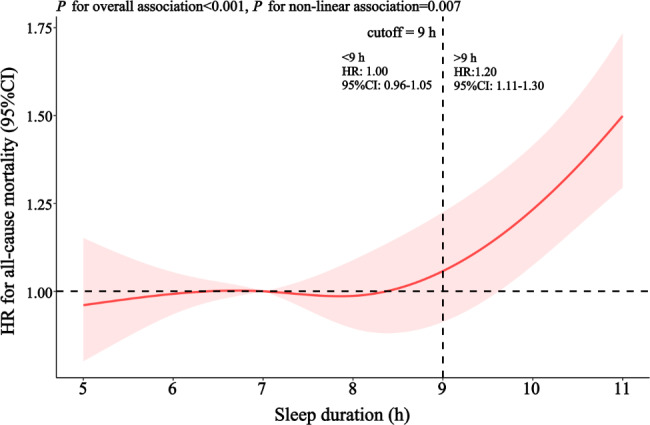
Spline curve for the association of sleep duration at baseline with all-cause mortality Adjusted for sex (male, female), age (continuous), place of residence (city, town, rural), education (illiteracy, educated), occupation (farmers, others), economic status (rich, general, poor), marital status (married, others), self-rated health (good, fair, poor), chronic disease (no, yes), depression (no, yes), cognitive function (normal, impairment), hypertension (no, yes), body type (underweight, normal, overweight), drinking status (never, former, current), smoking status (never, former, current), current exercise (no, yes), sleep quality (good, fair, poor), diet score (continuous)

Restrictive spline curves showed an inverse L-shaped association for sleep duration at baseline with risk of all-cause mortality, and threshold effect analysis showed that the best cut-off value was about 9 h, indicating a sharply increased risk of all-cause mortality in participants whose sleep duration exceeds 9 h (*P* for overall association < 0.001, *P* for non-linear association = 0.007) (Fig. 2).

## Discussion

### Main findings

In the current analysis, we found 5 trajectories of sleep duration in three consecutive surveys from 2005 to 2011 among the Chinese elderly. Compared with the persistent sleep trajectory of 7 h, the risk of all-cause mortality was higher in the moderately increased trajectory and rapidly increased trajectory, and the median survival time was shorter. These associations persisted after controlling for demographic characteristics, health-related factors, and lifestyle factors. Further, an inverse L-shaped association was found for sleep duration at a single baseline measurement with the risk of all-cause mortality, that is, when sleep duration exceeded 9 h, the risk of all-cause mortality increased dramatically. To the best of our knowledge, this was the first prospective study to explore the association of longitudinal sleep duration trajectories with the risk of all-cause mortality in an elderly population.

### Comparison with other studies

Conventional evidence supported a J-shaped association between sleep duration at a single baseline and all-cause mortality [19, 42], which suggested that long sleep (≥ 9 h) was associated with a higher risk of all-cause mortality than short sleep (≤ 6 h), possibly because short sleep was a normal physiological phenomenon in the elderly [43]. However, there are also studies reporting U-shaped associations [20], which may be related to confounding factors of unmeasured diseases or subclinical symptoms, and those studies did not fully account for the confounding of physical and mental health. Our study found the optimal threshold for single measured sleep duration and demonstrated an increased risk of all-cause mortality when sleep duration exceeded 9 h in the elderly after controlling for confounders. This provided new evidence to explore appropriate sleep durations for older adults and confirmed that long sleep (≥ 9-10 h) increases the risk of morbidity and mortality proposed by the American Academy of Sleep Medicine for older adults [6].

In our study, there were statistically significant differences in baseline characteristics between sleep trajectory groups. The older population in the increased sleep trajectory group was older, had a higher proportion of underweight body type, and had worse health outcomes. This was unsurprising and understandable, given that the increased sleep duration group was dominated by older adults who tend to have lower metabolisms, lower socioeconomic status, unhealthy diets, and leaner body types [17]. In addition, many studies suggest that increased sleep trajectory may be a marker of morbidity (hypertension, diabetes, depression, etc.) [6, 44], suggesting that older adults who reported increased sleep duration should be taken seriously.

Previous studies from the United States and Singapore with two waves of surveys reported that the *HR* (95%*CI*) of all-cause mortality for sleep duration increasing from 7 to 8 h to ≥ 9 h was 1.70 (1.30, 2.22) [19] and 1.43 (1.25, 1.64) [20], respectively, compared with persistent sleep duration of 7 h, while the risk of all-cause mortality was not significant when sleep duration decreased in the elderly population, which was consistent with our finding. We extended this finding in terms of longitudinal sleep duration trajectories, and our findings remained robust in stratified and sensitivity analyses. A study from China showed that decreased and persistent short sleep duration trajectories increased the risk of all-cause mortality [21], which contradicts our results, possibly because their participants were mostly middle-aged rather than elderly. This study complemented the evidence on the trajectory of increased sleep duration and risk of all-cause mortality in older adults from a life-course perspective of aging after controlling for confounders.

### Potential mechanisms

While the exact mechanisms through which sleep duration affects mortality may still be unclear, there are several potential pathways involved. Firstly, sleep duration in the elderly usually decreases naturally with age [22], so it is against the rules of healthy aging to sleep longer. This abnormal phenomenon may be associated with fatigue, decreased immune system, metabolic disorders, and inflammation [45–47], thus increasing the risk of a range of diseases such as cardiovascular disease and cancer, both of which are major causes of mortality. Secondly, long sleep duration has been shown to have negative effects on cognitive function [48], including memory, attention, and decision-making, which can lead to a range of health problems such as falls and injuries [49], and ultimately may lead to premature mortality. Thirdly, there is much evidence that long sleep duration may affect mental and emotional health [47, 50], where stress and dysfunctional hypothalamic-pituitary-adrenal axis activity may ultimately lead to unintentional injuries and an increased risk of mortality [51]. Finally, Abnormal melatonin secretion in older adults with long sleep duration may also have high daily sleep variability [52, 53], disrupting biorhythmic homeostasis and further reducing anti-inflammatory capacity [54, 55], so this feedback can further induce a variety of chronic diseases (e.g. depression, cognitive impairment, diabetes, stroke) and even mortality [56–59], which may be another potential mechanism. These mechanisms suggest that this increased sleep duration trajectory in the elderly is an important marker of abnormal aging and should be alerted.

### Strengths and limitations

Strengths of this study include a prospective cohort, longitudinal follow-up, large sample size, fully adjusted covariates, and modeling using a longitudinal sleep duration trajectory approach. Likewise, several limitations also exist in our study. Firstly, sleep duration was self-reported, but obtaining objective and detailed sleep duration was often not feasible in large prospective studies. Studies have shown that self-reported sleep duration is moderately correlated with objective measures (e.g., sleep logs, polysomnography) [31, 60], indicating that self-reported sleep duration can also be used in epidemiological studies. Secondly, as the CLHLS database does not contain variables for daytime napping and nighttime sleep, it limits our further research. Thirdly, although we excluded people who died within two years of follow-up and the trend of association was consistent, the follow-up period for this study was short (2011–2019; only 8 years), so reverse causality may still be inevitable. Fourthly, the participants in this study were Chinese elderly, and the findings cannot be generalizable to young adults and other ethnic populations. Finally, although we included as many covariates as possible in the multivariate-adjusted model, confounding variables such as sleep disturbance (e.g., sleep apnea, sleep disruption, insomnia), medication use, fatigue, and stress were not collected, and the interpretation of the results should be cautious. Our study supplements the evidence at an advanced age and provides a basis for guiding healthy sleep in the elderly. In future studies, we should obtain as many dimensions of sleep behavior as possible to more scientifically explain the association between long-term sleep duration trajectories and all-cause mortality.

## Conclusions

Our study found that, unlike the middle-aged population, in Chinese older adults, compared with persistent sleep trajectory of 7 h, moderately increased trajectory and rapidly increased trajectory were both associated with shortened survival time and a higher risk of all-cause mortality. Combined with previous studies, it should be taken seriously when older adults show signs of increased sleep duration, especially when sleep duration exceeded 9 h. Our study enriches the use of sleep duration trajectories in health outcomes and also provides a new view of all-cause mortality research. In the future, our study suggests that health workers should improve sleep health education for older people, especially when they report increased sleep duration trajectory or long sleep duration. In addition, the government and the community should pay close attention to the changing regularities of sleep duration in the elderly population and conduct better sleep behavior cohort studies in the future to suggest appropriate sleep duration for the elderly.

## Electronic supplementary material

Below is the link to the electronic supplementary material.

Supplementary Material 1

## Data Availability

All data used in this study were accessed from the publicly available Chinese Longitudinal Healthy Longevity Survey (https://opendata.pku.edu.cn/dataverse/CHADS). The datasets used are available from the corresponding author on reasonable request.

## References

[1] United Nations. : The world’s population is ageing and social security should be reconsidered. Jan 12, 2023 https://news.un.org/zh/story/2023/01/1114127.

[2] China Yearly Statistics. : About the China Data Institute Datasets. Jan 27, 2023 https://data-planet.libguides.com/CDC.

[3] Hall EW, Vaughan AS, Ritchey MD (2019). Stagnating National declines in stroke mortality mask widespread County-Level increases, 2010–2016. Stroke.

[4] Smittenaar CR, Petersen KA, Stewart K (2016). Cancer incidence and mortality projections in the UK until 2035. Br J Cancer.

[5] Besedovsky L, Lange T, Haack M (2019). The Sleep-Immune Crosstalk in Health and Disease. Physiol Rev.

[6] Hirshkowitz M, Whiton K, Albert SM (2015). National Sleep Foundation’s updated sleep duration recommendations: final report. Sleep Health.

[7] Wang S, Li B, Wu Y (2017). Relationship of Sleep Duration with Sociodemographic characteristics, Lifestyle, Mental Health, and chronic Diseases in a large Chinese Adult Population. J Clin Sleep Med.

[8] Sabia S, Dugravot A, Léger D (2022). Association of sleep duration at age 50, 60, and 70 years with risk of multimorbidity in the UK: 25-year follow-up of the Whitehall II cohort study. PLoS Med.

[9] Wang S, Wu Y, Ungvari GS (2017). Sleep duration and its association with demographics, lifestyle factors, poor mental health and chronic diseases in older chinese adults. Psychiatry Res.

[10] He L, Biddle SJH, Lee JT (2021). The prevalence of multimorbidity and its association with physical activity and sleep duration in middle aged and elderly adults: a longitudinal analysis from China. Int J Behav Nutr Phys Act.

[11] Ruiz-Castell M, Makovski TT, Bocquet V (2019). Sleep duration and multimorbidity in Luxembourg: results from the European Health Examination Survey in Luxembourg, 2013–2015. BMJ Open.

[12] Loprinzi PD (2015). Health-enhancing multibehavior and medical multimorbidity. Mayo Clin Proc.

[13] Cappuccio FP, D’Elia L, Strazzullo P (2010). Sleep duration and all-cause mortality: a systematic review and meta-analysis of prospective studies. Sleep.

[14] Ohayon MM, Carskadon MA, Guilleminault C (2004). Meta-analysis of quantitative sleep parameters from childhood to old age in healthy individuals: developing normative sleep values across the human lifespan. Sleep.

[15] Baden MY, Hu FB, Vetter C (2020). Sleep duration patterns in early to Middle Adulthood and subsequent risk of type 2 diabetes in women. Diabetes Care.

[16] Chen J, Patel SR, Redline S, et al. Weekly sleep trajectories and their associations with obesity and hypertension in the Hispanic/Latino population. Sleep. 2018;41(10). 10.1093/sleep/zsy150. 10.1093/sleep/zsy150 PMC6187108 30053253

[17] Yi SJ, Jeong YM, Kim JH. Relationship between Sleep Duration Trajectories and Self-Rated depressive symptoms in South Koreans with Physical Disabilities. Healthc (Basel). 2021;9(3). 10.3390/healthcare9030361. 10.3390/healthcare9030361 PMC8005137 33806836

[18] Cheng GH, Malhotra R, Østbye T, et al. Changes in nocturnal sleep and daytime nap durations predict all-cause mortality among older adults: the panel on Health and Ageing of Singaporean Elderly. Sleep. 2018;41(7). 10.1093/sleep/zsy087. 10.1093/sleep/zsy087 29722881

[19] Aurora RN, Kim JS, Crainiceanu C (2016). Habitual sleep duration and all-cause mortality in a General Community Sample. Sleep.

[20] Soh AZ, Chee MWL, Yuan JM, et al. Sleep lengthening in late adulthood signals increased risk of mortality. Sleep. 2018;41(3). 10.1093/sleep/zsy005. 10.1093/sleep/zsy005 PMC5914333 29394410

[21] Wang YH, Wang J, Chen SH (2020). Association of longitudinal patterns of Habitual Sleep Duration with Risk of Cardiovascular events and all-cause mortality. JAMA Netw Open.

[22] Chylinski D, Van Egroo M, Narbutas J, et al. Timely coupling of sleep spindles and slow waves linked to early amyloid-β burden and predicts memory decline. Elife. 2022;11. 10.7554/eLife.78191. 10.7554/eLife.78191 PMC9177143 35638265

[23] Ren Y, Miao M, Yuan W (2020). Sleep duration and all-cause mortality in the elderly in China: a population-based cohort study. BMC Geriatr.

[24] Shi Z, Zhang T, Byles J (2015). Food Habits, Lifestyle factors and mortality among Oldest Old Chinese: the chinese longitudinal healthy longevity survey (CLHLS). Nutrients.

[25] Ji JS, Zhu A, Bai C (2019). Residential greenness and mortality in oldest-old women and men in China: a longitudinal cohort study. Lancet Planet Health.

[26] Yan LL, Li C, Zou S (2022). Healthy eating and all-cause mortality among chinese aged 80 years or older. Int J Behav Nutr Phys Act.

[27] Ruan R, Feng L, Li J (2013). Tea consumption and mortality in the oldest-old chinese. J Am Geriatr Soc.

[28] Gu D, Sautter J, Pipkin R (2010). Sociodemographic and health correlates of sleep quality and duration among very old chinese. Sleep.

[29] Cavaillès C, Carrière I, Wagner M, et al. Trajectories of sleep duration and timing before dementia: a 14-year follow-up study. Age Ageing. 2022;51(8). 10.1093/ageing/afac186. 10.1093/ageing/afac186 35977152

[30] Liu TZ, Xu C, Rota M (2017). Sleep duration and risk of all-cause mortality: a flexible, non-linear, meta-regression of 40 prospective cohort studies. Sleep Med Rev.

[31] Lauderdale DS, Knutson KL, Yan LL (2008). Self-reported and measured sleep duration: how similar are they?. Epidemiology.

[32] Ding P, Li J, Chen H (2022). Independent and joint effects of sleep duration and sleep quality on suboptimal self-rated health in medical students: a cross-sectional study. Front Public Health.

[33] Lockley SW, Skene DJ, Arendt J (1999). Comparison between subjective and actigraphic measurement of sleep and sleep rhythms. J Sleep Res.

[34] Bai C, Guo M, Yao Y (2021). Sleep duration, vegetable consumption and all-cause mortality among older adults in China: a 6-year prospective study. BMC Geriatr.

[35] Wu S, Lv X, Shen J (2021). Association between body mass index, its change and cognitive impairment among chinese older adults: a community-based, 9-year prospective cohort study. Eur J Epidemiol.

[36] Tey NP, Lai SL, Teh JK (2016). The debilitating effects of chronic diseases among the oldest old in China. Maturitas.

[37] Zhou M, Wang H, Zeng X (2019). Mortality, morbidity, and risk factors in China and its provinces, 1990–2017: a systematic analysis for the global burden of Disease Study 2017. Lancet.

[38] Lv X, Li W, Ma Y (2019). Cognitive decline and mortality among community-dwelling chinese older people. BMC Med.

[39] Zhang Y, Xiong Y, Yu Q (2021). The activity of daily living (ADL) subgroups and health impairment among chinese elderly: a latent profile analysis. BMC Geriatr.

[40] Gilmour H, Stranges S, Kaplan M (2013). Longitudinal trajectories of sleep duration in the general population. Health Rep.

[41] Zhang M, Lv X, Chen Y (2022). Excessive sleep increased the risk of incidence of cognitive impairment among older chinese adults: a cohort study based on the chinese longitudinal healthy longevity survey (CLHLS). Int Psychogeriatr.

[42] Kwok CS, Kontopantelis E, Kuligowski G (2018). Self-reported Sleep Duration and Quality and Cardiovascular Disease and Mortality: a dose-response Meta-analysis. J Am Heart Assoc.

[43] Scullin MK, Bliwise DL (2015). Sleep, cognition, and normal aging: integrating a half century of multidisciplinary research. Perspect Psychol Sci.

[44] Huang L, Long Z, Lyu J (2021). The Associations of Trajectory of Sleep Duration and inflammation with hypertension: a longitudinal study in China. Nat Sci Sleep.

[45] Mander BA, Winer JR, Walker MP (2017). Sleep and human aging. Neuron.

[46] Swift CG, Shapiro CM (1993). ABC of sleep disorders. Sleep and sleep problems in elderly people. BMJ.

[47] Prather AA, Vogelzangs N, Penninx BW (2015). Sleep duration, insomnia, and markers of systemic inflammation: results from the Netherlands Study of Depression and anxiety (NESDA). J Psychiatr Res.

[48] Ma Y, Liang L, Zheng F (2020). Association between Sleep Duration and Cognitive decline. JAMA Netw Open.

[49] Fuller GF (2000). Falls in the elderly. Am Fam Physician.

[50] Dong L, Xie Y, Zou X (2022). Association between sleep duration and depression in US adults: a cross-sectional study. J Affect Disord.

[51] O’Connor DB, Gartland N, O’Connor RC (2020). Stress, cortisol and suicide risk. Int Rev Neurobiol.

[52] Huang T, Mariani S, Redline S (2020). Sleep Irregularity and Risk of Cardiovascular events: the multi-ethnic study of atherosclerosis. J Am Coll Cardiol.

[53] Huang T, Redline S (2019). Cross-sectional and prospective Associations of Actigraphy-Assessed sleep regularity with metabolic abnormalities: the multi-ethnic study of atherosclerosis. Diabetes Care.

[54] Mattis J, Sehgal A (2016). Circadian rhythms, Sleep, and Disorders of Aging. Trends Endocrinol Metab.

[55] Hardeland R, Aging. Melatonin, and the Pro- and anti-inflammatory networks. Int J Mol Sci. 2019;20(5). 10.3390/ijms20051223. 10.3390/ijms20051223 PMC6429360 30862067

[56] Cantarero-Prieto D, Pascual-Sáez M, Blázquez-Fernández C (2018). Social isolation and multiple chronic diseases after age 50: a european macro-regional analysis. PLoS ONE.

[57] Vaiserman AM, Koliada AK, Marotta F (2017). Gut microbiota: a player in aging and a target for anti-aging intervention. Ageing Res Rev.

[58] Zhou L, Yu K, Yang L (2020). Sleep duration, midday napping, and sleep quality and incident stroke: the Dongfeng-Tongji cohort. Neurology.

[59] Svensson T, Saito E, Svensson AK (2021). Association of Sleep Duration with All- and major-cause mortality among adults in Japan, China, Singapore, and Korea. JAMA Netw Open.

[60] Patel SR, Ayas NT, Malhotra MR (2004). A prospective study of sleep duration and mortality risk in women. Sleep.

