# Supplementary material for: Sleep duration trajectories and all-cause mortality among Chinese elderly: A community-based cohort study
Source: BMC Public Health. 2023 Jun 6;23:1095. doi: 10.1186/s12889-023-15894-3 (PMC10286431; doi:10.1186/s12889-023-15894-3)

**Supplementary material**

**Sleep duration trajectories and all-cause mortality among Chinese elderly: a community-based cohort study**

Rongxiu Ding, Pan Ding, Liuhong Tian, Xiaodan Kuang, Li Huang, Hongying Shi*

**eTable 1.** Baseline characteristics by exclusions

**eTable 2** Fit indices of growth mixed modeling on sleep duration trajectories

**eTable 3** Association between sleep duration trajectories and the risk of all-cause mortality, stratification analyses for health status

**eFig. 1** Flow chart of the study population, a prospective cohort (2005-2018)

**eFig. 2** Distribution of sleep duration in three waves of follow-up survey

**eFig. 3** Kaplan-Meier survival curves for participants with different trajectories of sleep duration

**eTable 1 Baseline characteristics by exclusions**

| **Characteristics** | **Included (*n*=3895)** | **Excluded (*n*=240)** | ***P*-value** |
| --- | --- | --- | --- |
| **Age, years, median (*IQR*)** | 82.0 (76.0, 91.0) | 82.0 (76.0, 91.0) | 0.249 |
| **Female, *n* (%)** | 2077 (53.3) | 133 (55.4) | 0.573 |
| **Married, *n* (%)** | 1616 (41.5) | 94 (39.2) | 0.521 |
| **Farmers, *n* (%)** | 2572 (66.0) | 98 (40.8) | **<0.001** |
| **Illiteracy, *n* (%)** | 2087 (53.6) | 111 (46.3) | **0.032** |
| **Economic status, *n* (%)** |  |  | 0.717 |
| Rich | 711 (18.3) | 39 (16.3) |  |
| General | 2497 (64.1) | 159 (66.3) |  |
| Poor | 687 (17.6) | 42 (17.5) |  |
| **Body type, *n* (%)** |  |  | 0.317 |
| Underweight | 1053 (27.0) | 58 (24.2) |  |
| Normal | 2078 (53.4) | 126 (52.5) |  |
| Overweight | 764 (19.6) | 56 (23.3) |  |
| **Sleep quality, *n* (%)** |  |  | 0.320 |
| Good | 2404 (61.7) | 149 (62.1) |  |
| Fair | 897 (23.0) | 62 (25.8) |  |
| Poor | 594 (15.3) | 29 (12.1) |  |
| **Diet score, median (*IQR*)** | 12.0 (10.0, 15.0) | 13.0 (10.0, 15.0) | 0.258 |
| **Drink, *n* (%)** |  |  | **0.028** |
| Never | 2481 (63.7) | 168 (70.0) |  |
| Former | 678 (17.4) | 43 (17.9) |  |
| Current | 736 (18.9) | 29 (12.1) |  |
| **Smoke, *n* (%)** |  |  | **0.007** |
| Never | 2394 (61.5) | 150 (62.5) |  |
| Former | 743 (19.1) | 60 (25.0) |  |
| Current | 758 (19.5) | 30 (12.5) |  |
| **Current exercise, *n* (%)** | 1613 (41.4) | 102 (42.5) | 0.218 |
| **Self-rated health, *n* (%)** |  |  | 0.806 |
| Good | 1,726 (44.3) | 106 (44.2) |  |
| Fair | 1,388 (35.6) | 82 (34.2) |  |
| Poor | 781 (20.1) | 52 (21.7) |  |
| **Cognitive impairment, *n* (%)** | 1,414 (36.3) | 101 (42.1) | 0.083 |
| **Have Depression, *n* (%)** | 1,519 (39.0) | 96 (40.0) | 0.810 |
| **Have chronic disease, *n* (%)** | 1,518 (39.0) | 108 (45.0) | 0.074 |
| **Hypertension, *n* (%)** | 1,539 (39.5) | 92 (38.3) | 0.768 |

The Chi-square test was used for unordered categorical data, the Wilcoxon rank sum test was used for ordinal data and skewed continuous data, and bold values indicated statistical significance *P*<0.05.

**eTable 2 Fit indices of growth mixture modeling on sleep duration trajectories**

| **Model** | **Log-likelihood** | **AIC** | **BIC** | **aBIC** | **Entropy** | **Class (%)** |
| --- | --- | --- | --- | --- | --- | --- |
| 1 | -23441.42 | 46902.85 | 46965.60 | 46933.83 | 1.00 | 100.0 |
| 2 | -23216.45 | 46460.90 | 46548.76 | 46504.27 | 0.662 | 57.0/43.0 |
| 3 | -22756.51 | 45549.02 | 45661.98 | 45604.78 | 0.898 | 26.9/41.9/31.2 |
| 4 | -23143.48 | 46330.96 | 46469.02 | 46399.11 | 0.615 | 17.1/38.5/14.6/29.8 |
| 5 | -22730.76 | 45513.52 | 45676.67 | **45594.06** | 0.773 | 28.1/7.2/33.7/21.3/9.7 |
| 6 | -22730.76 | 45521.51 | 45709.77 | 45614.45 | 0.745 | 0.0/17.4/4.9/9.9/26.2/41.6 |

Notes: AIC, Akaike Information Criterion; BIC, Bayesian Information Criterion; aBIC, adjusted Bayesian Information Criterion.

**eTable 3 Association between sleep duration trajectories and the risk of all-cause mortality, stratification analyses for health status**

| **Subgroups** | ***n*** | **Cases/person-years** | **Sleep duration trajectories, **HRs* (95%*CI*) *P*-value** | | | | | ***P* for interaction** |
| --- | --- | --- | --- | --- | --- | --- | --- | --- |
|  |  |  | **Moderately increased** | **Rapidly increased** | **Persistent sleep** | **Moderately decreased** | **Rapidly decreased** |  |
| **Self-rated health** |  |  |  |  |  |  |  | 0.366 |
| Good | 1720 | 757/7670 | **1.39 (1.16, 1.66) <0.001** | 1.19 (0.90, 1.59) 0.208 | 1.00 | 0.82 (0.64, 1.05) 0.109 | 0.93 (0.69, 1.25) 0.630 |  |
| Fair | 1386 | 657/5981 | **1.29 (1.06, 1.57) 0.012** | 1.35 (0.99, 1.84) 0.054 | 1.00 | 0.99 (0.78, 1.27) 0.943 | 0.91 (0.66, 1.25) 0.542 |  |
| Poor | 789 | 467/3039 | 1.26 (0.97, 1.65) 0.086 | 1.38 (0.97, 1.97) 0.074 | 1.00 | 0.80 (0.61, 1.06) 0.122 | 0.85 (0.61, 1.18) 0.325 |  |
| **Cognitive function** |  |  |  |  |  |  |  | 0.199 |
| Normal | 2481 | 907/11997 | **1.27 (1.07, 1.50) 0.006** | 1.12 (0.86, 1.47) 0.396 | 1.00 | 0.93 (0.76, 1.13) 0.453 | 1.00 (0.77, 1.28) 0.977 |  |
| Impairment | 1414 | 974/4692 | **1.34 (1.14, 1.59) <0.001** | **1.42 (1.11, 1.80) 0.004** | 1.00 | 0.82 (0.66, 1.02) 0.082 | 0.79 (0.62, 1.02) 0.073 |  |
| **Depression** |  |  |  |  |  |  |  | 0.162 |
| No | 2376 | 951/11091 | **1.25 (1.06, 1.48) 0.007** | 1.14 (0.88, 1.48) 0.308 | 1.00 | 0.92 (0.75, 1.12) 0.417 | 0.98 (0.74, 1.28) 0.861 |  |
| Yes | 1519 | 930/5598 | **1.23 (1.03, 1.45) 0.019** | 1.23 (0.96, 1.58) 0.095 | 1.00 | 0.86 (0.70, 1.07) 0.175 | 0.81 (0.64, 1.03) 0.083 |  |
| **Chronic disease** |  |  |  |  |  |  |  | 0.143 |
| No | 2377 | 1121/10546 | **1.21 (1.04, 1.41) 0.014** | **1.32 (1.05, 1.67) 0.018** | 1.00 | 0.94 (0.78, 1.14) 0.559 | 0.90 (0.72, 1.13) 0.362 |  |
| Yes | 1518 | 760/6143 | **1.28 (1.06, 1.54) 0.010** | 1.09 (0.83, 1.45) 0.530 | 1.00 | **0.76 (0.61, 0.95) 0.017** | 0.83 (0.62, 1.11) 0.200 |  |
| **Hypertension** |  |  |  |  |  |  |  | 0.248 |
| No | 2356 | 1176/9997 | **1.34 (1.15, 1.55) <0.001** | **1.39 (1.11, 1.73) 0.003** | 1.00 | 0.86 (0.71, 1.04) 0.124 | 0.90 (0.73, 1.12) 0.364 |  |
| Yes | 1539 | 705/6692 | **1.32 (1.09, 1.60) 0.005** | 1.11 (0.82, 1.50) 0.508 | 1.00 | 0.91 (0.72, 1.15) 0.424 | 0.89 (0.66, 1.21) 0.469 |  |

Abbreviation: HR, hazard ratio; CI, confidence interval.

Moderately increased trajectory (from 8 to 10h), rapidly increased trajectory (from 6 to 9h), moderately decreased trajectory (from 7 to 5h), rapidly decreased trajectory (from 8 to 5h).

*Adjusted for sex (male, female), age (≤80, >80 years), place of residence (city, town, rural), education (illiteracy, educated), occupation (farmers, others), economic status (rich, general, poor), marital status (married, others), self-rated health (good, fair, poor), chronic disease (no, yes), depression (no, yes), cognitive function (normal, impairment), activities of daily living (normal, disabled), hypertension (no, yes), body type (underweight, normal, overweight), drinking status (never, former, current), smoking status (never, former, current), current exercise (no, yes), sleep quality (good, fair, poor), diet score (continuous), except for the stratification factor itself. And bold values indicated statistical significance *P*<0.05.

**eFig. 1 Flow chart of the study population, a prospective cohort (2005-2018)**

Participants surveyed in 2005, 2008 and 2011, *n*=4,191

The remaining participants were included in the study, *n*=3,895

Exclude those lost to follow-up without any follow-up records and no date of death, *n*=240

Exclude those who missed sleep duration variables in the 2005, 2008 or 2011 surveys, *n*=56

Participants included in the follow-up, n=4,135

**eFig. 2 Distribution of sleep duration in three waves of follow-up survey**


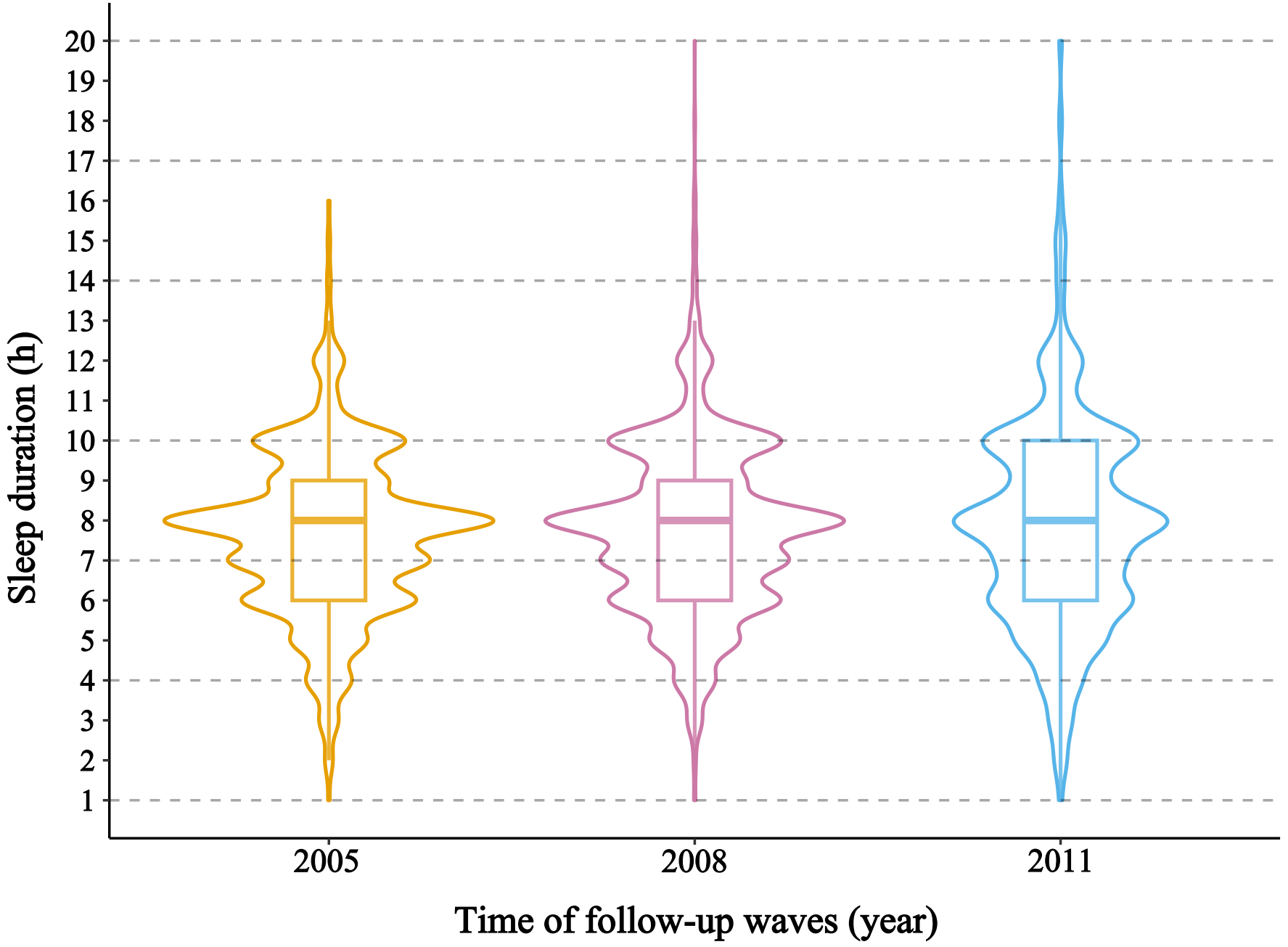


**eFig. 3 Kaplan-Meier survival curves for participants with different trajectories of sleep duration**


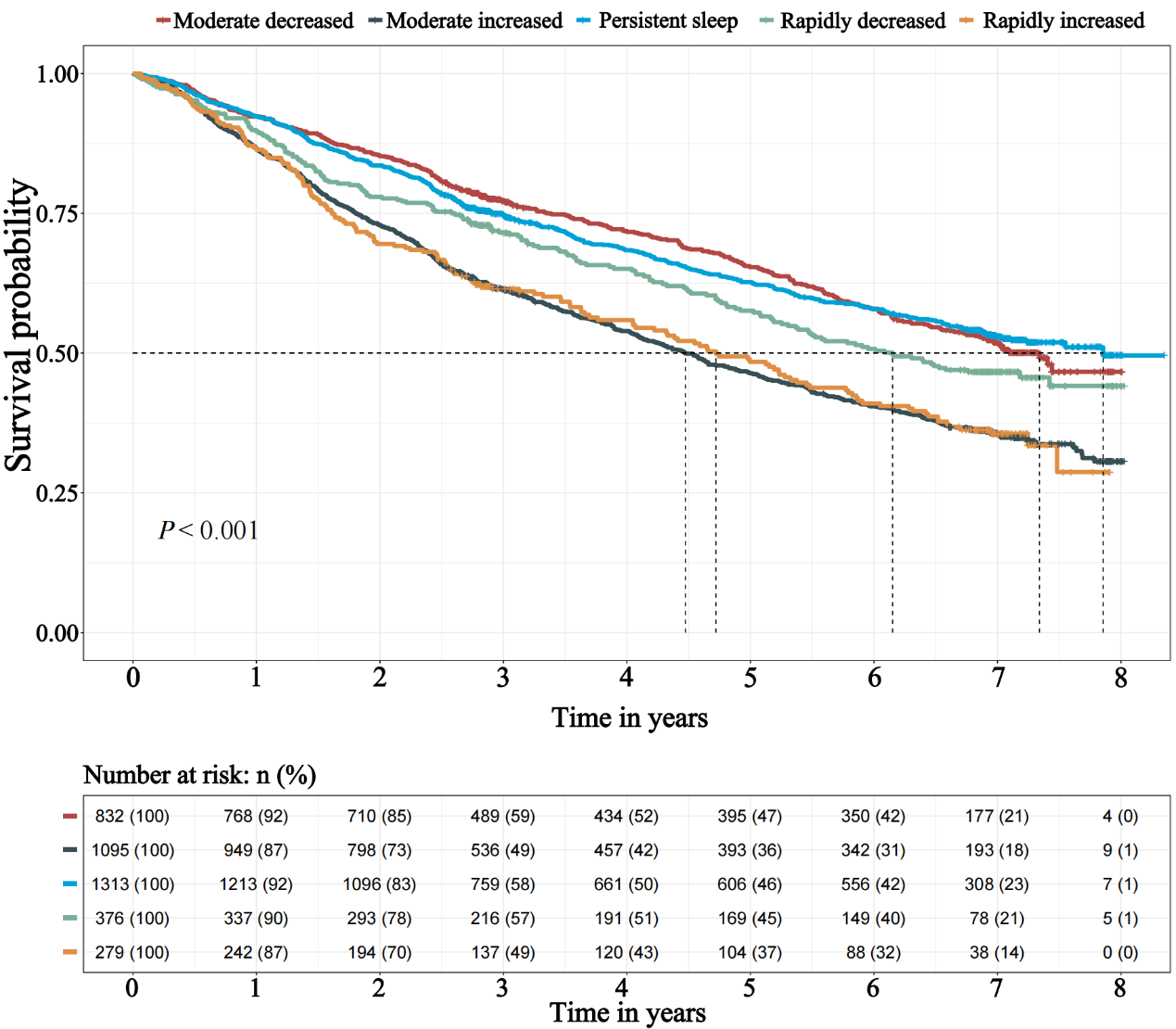

Supplement: Supplementary file 1 — Supplementary Material 1 [file 12889_2023_15894_MOESM1_ESM.docx]
